# Analysis of risk stratification and prevention of sudden death in pediatric patients with hypertrophic cardiomyopathy: Dilemmas and clarity

**DOI:** 10.1016/j.hroo.2023.06.007

**Published:** 2023-06-20

**Authors:** Jiri Bonaventura, Barry J. Maron, Charles I. Berul, Ethan J. Rowin, Martin S. Maron

**Affiliations:** ∗ Department of Cardiology, 2nd Faculty of Medicine, Charles University and Motol University Hospital, Prague, Czech Republic; † Hypertrophic Cardiomyopathy Center, Lahey Hospital and Medical Center, Burlington, Massachusetts; ‡ Division of Cardiology, Children’s National Hospital, Department of Pediatrics, George Washington University School of Medicine, Washington, DC

**Keywords:** Hypertrophic cardiomyopathy, Sudden death, Implantable cardioverter-defibrillators, Ventricular arrhythmias, Decision making, Pediatric

## Abstract

Hypertrophic cardiomyopathy (HCM) has been considered the most common cause of sudden death (SD) in the young. However, introduction of implantable cardioverter-defibrillators (ICDs) in HCM has proved highly effective and the mainstay of preventing SD in children, adolescents, and adults by terminating malignant ventricular tachyarrhythmias. Nevertheless, ICD decision making is generally regarded as more difficult in pediatrics, and the strategy for selecting ICD patients from this population remains without consensus. Prospective studies in HCM children and adolescents have shown the American Heart Association/American College of Cardiology traditional major risk marker strategy to be reliable with >90% sensitivity in selecting patients for SD prevention. International data in >2000 young HCM patients assembled over 20 years who were stratified by major risk markers showed ICDs effectively prevented SD in 20%. Alternatively, novel quantitative risk scoring initiatives provide 5-year risk estimates that are potentially useful as adjunctive tools to facilitate discussion of prophylactic ICD risks vs benefit but are as yet unsupported by prospective outcome studies. Risk scoring strategies are characterized by reasonable discriminatory statistical power (C-statistic 0.69-0.76) for identifying patients with SD events but with relatively low sensitivity, albeit with specificity comparable with the risk marker strategy. While some reticence for obligating healthy-appearing young patients to lifelong device implants is understandable, underutilization of the ICD in high-risk children and adolescents can represent a lost opportunity for fulfilling the long-standing aspiration of SD prevention. This review provides a critical assessment of the current strengths and weaknesses of SD risk stratification strategies in young HCM patients in an effort to clarify clinical decision making in this challenging subpopulation.

**Key Findings**

▪ Recommending prophylactic implantable cardioverter-defibrillators to pediatric hypertrophic cardiomyopathy patients is a unique clinical situation and among the most difficult management decisions in cardiology.
▪ The American Heart Association/American College of Cardiology major risk marker strategy has proved highly sensitive in predicting arrhythmic sudden death events and utilizing the power of primary prevention implantable cardioverter-defibrillators to prevent sudden death in young hypertrophic cardiomyopathy patients.
▪ Quantitative risk scoring initiatives provide short-term estimates for sudden death events, potentially useful in patient-physician interactions by offering additional information relevant to primary prevention decision making.
▪ Prediction of sudden death outcome by risk scores in pediatrics is not yet supported by prospective study data.
▪ Modest overtreatment with implantable cardioverter-defibrillators associated with current risk stratification algorithms represents a future investigative priority to improve patient selection.

## Introduction

In patients with hypertrophic cardiomyopathy (HCM), sudden death (SD) can occur at virtually any age, with these tragic events often the first manifestation of the disease.^1^ Indeed, longitudinal datasets have demonstrated that in children with HCM, arrhythmic events are responsible for a majority of adverse events occurring within 10 years of diagnosis.^2^

Notably, prevention of arrhythmic SD with implantable cardioverter-defibrillators (ICDs) has been a reliable lifesaving therapy in high-risk HCM patients of all ages, including children and adolescents.^3^ Accurate identification of those patients deserving of ICDs requires predicting for a stable individual patient an isolated arrhythmic event anticipated to occur at some unknown time in the future.

The available literature on risk stratification and ICD decision making for young patients can at times seem overwhelming and potentially confusing to practicing clinicians entrusted with the management of these patients. Whether to recommend an ICD as primary prevention entails complex clinical logic to balance the procedural risks and device-related complications against the long-term arrhythmic risk. Notably, ICDs are fraught with long-standing maintenance issues and potential device-related complications (eg, inappropriate shocks, lead failures, and multiple device replacements occurring over decades), as well as possibly imposing selective restrictions on quality of life.

Accurate identification of children with HCM at highest risk who would benefit most from ICDs (while minimizing risk of complications) is paramount. The present commentary summarizes the relevant issues to date in a balanced fashion providing a measure of clarity and potential solutions to this important and difficult clinical problem in the underrecognized subpopulation of pediatric HCM patients. Nevertheless, many questions remain that benefit from discussion of the varying perspectives represented in this clinical arena.

## General principles and issues regarding ICDs in children

Recommending prophylactic ICDs to particularly young HCM patients is a unique clinical situation, arguably among the most difficult management decisions in cardiology, and generally more complex than encountered in adults with this disease. Indeed, it is paradoxical that while ICD decisions for lifesaving treatment may be relatively uncommon in young HCM patients, this is nevertheless an age group with a certain predilection for SD.^4^

Young high-risk HCM patients usually appear in excellent health without symptoms and often show interest for engaging in a vigorous lifestyle including a variety of sports. Furthermore, there has been uncertainty among the pediatric cardiology community whether the risk stratification procedure for children with HCM should follow the same methodology as that employed in adults, with perceived gaps in extrapolation. Indeed, some authors are concerned that the predictors of adverse outcomes in children may differ from those of adults with HCM.^5^^,^^6^ Also, conditions associated with left ventricular (LV) hypertrophy that mimic HCM (ie, phenocopies) are more frequently encountered in childhood. It is recently reported that RASopathies have higher incidence of nonarrhythmic death or transplantation but similar incidence of SD as HCM, albeit ICDs were less frequently used.^7^

These concerns are coupled with the technical challenges of device implantation in smaller and growing patients and a higher incidence of device-related complications reported in pediatrics, including (1) inappropriate shocks (due to sinus and supraventricular tachycardia, or T-wave oversensing), (2) infections, (3) lead fractures, (4) required multiple generator replacements, (5) the possibility for lead extractions over many decades of life, (6) thrombosis and venous occlusion,^8^ and (7) potential requirement for implantation with epicardial, pleural, and pericardial patch or coil-based systems^9^ as alternatives to traditional transvenous leads.^10^

There is an early experience with subcutaneous lead systems in young patients, although enthusiasm for this option is restrained by large bulky generators and absent pacing capability.^11^, ^12^, ^13^ Nevertheless, ICD implants in children are likely to be lifelong, given that HCM is a disease compatible with normal life expectancy, and associated with unpredictable delays in timing of therapeutic interventions, eg, ≥ 10 years in one-third of patients following implantation.^14^

Recent studies showed that the frequency of inappropriate shocks has decreased in patients of all ages due to advances in device programming, including longer detection times and rate criteria tailored to patient age, clinical history, and level of activity.^15^^,^^16^ While device complications are unpredictable,^14^ truly long-term rates are unknown in pediatric populations.^17^ It should be expected, however, that pediatric patients implanted with an ICD for primary prevention will live many decades after implantation, necessitating multiple generator replacements and possible eventual lead fracture or failure requiring extraction and replacement, with the known attendant risks of these procedures.

## Principles of risk stratification

ICDs have proven remarkably reliable in terminating potentially lethal ventricular tachyarrhythmias in HCM patients of all ages and body sizes. However, given the challenge of predicting isolated arrhythmic events in individual patients over long periods of time, areas of uncertainty persist concerning proper selection of young patients for prophylactic implants, encumbered by several factors: (1) data are presented from relatively small retrospective studies encompassing heterogeneous populations and often including syndromic conditions^4^^,^^7^^,^^18^ associated with LV hypertrophy that mimic HCM (ie, phenocopies such as LAMP2 cardiomyopathy, PRKAG2 cardiomyopathy, or Noonan syndrome), (2) lack of consensus among authors regarding the definitions of some high-risk markers, and (3) unresolved concern among some investigators that individual risk markers have limited sensitivity and specificity to predict sudden cardiac death (SCD) in children.

The major risk factors most frequently associated with high sensitivity for identifying pediatric patients at greatest SD risk in longitudinal ICD studies, largely based on American Heart Association/American College of Cardiology (AHA/ACC) major risk markers^8^^,^^19^^,^^20^ are (1) extreme LV hypertrophy ≥30 mm (or closely approaching this arbitrary cutpoint), independent of body size, (2) ≥1 unexplained syncopal episode, (3) family history of≥1 HCM-related SDs, and (4) repetitive or prolonged nonsustained ventricular tachycardia on ambulatory monitoring.

These markers overlap with those used conventionally to assess SD risk in adult HCM patients. In addition, other risk markers in adult HCM patients are reasonable to consider as possibilities in children, albeit less common in this age group and supported with little specific data (eg, LV apical aneurysm, extensive late gadolinium enhancement by cardiac magnetic resonance, systolic dysfunction [ejection fraction<50%]).^21^ Prospective assessment of these latter markers has not been adequately performed in large-scale pediatric HCM populations. There is general agreement that sarcomere variants, whether in patients with or without phenotype expression (ie, LV hypertrophy), have little prognostic value^21^ and consequently have no reliable role in risk stratification.

Some SD risk factors have not achieved standard definitions in the published literature, which has created a measure of confusion for risk stratification. For example, although there is general agreement that greater ventricular septal hypertrophy is directly related to likelihood of SD events, the method for defining the magnitude of LV wall thickness remains unsettled in pediatrics. Traditionally, investigators have used the binary marker of absolute LV thickness independent of age and body size. In fact, appropriate ICD therapy most often occurs in pediatric patients who have maximal LV wall thickness at, near, or beyond the proposed ≥30-mm cutpoint.^19^

As an alternative, the pediatric cardiology community has emphasized the importance of normalizing echocardiographic parameters to body size, such as LV hypertrophy, relying on the calculated *Z* score to adjust LV wall thickness to body surface area and growth as a continuous variable.^22^^,^^23^ However, at present, no consensus has emerged regarding the binary *Z* score threshold cutoff values that are aligned with increased SD risk^23^ and could serve as independent risk markers guiding clinical practice and ICD decision making; there are no specific *Z* score data related directly to ICD performance.

On a cautionary note, using the *Z* score as a sole risk marker could lead to excessive implantations in young patients with relatively mild absolute LV wall thickness. For example, a *Z* score of ≥6 has been regarded as equivalent to massive LV hypertrophy,^19^ but in most pediatric cohorts ICD implantation based on this particular *Z* score alone has not been linked to appropriate device therapy.^19^^,^^20^^,^^24^^,^^25^ Furthermore, notably a variety of *Z* scores have been advanced in independent populations using mathematically different models,^23^ delivering different scores for the same patients.

A risk marker that frequently appears in the pediatric literature is a family history of HCM-related SD. Historically, varying definitions for this risk factor have been introduced in the literature as well as in European and North American guidelines.^26^, ^27^, ^28^ For example, some studies have advocated ≥2 familial HCM-related SDs, whereas others have considered events in only 1 relative, or give greater weight to SDs occurring in only first-degree relatives or relatives <50 years of age.^26^, ^27^, ^28^

Nevertheless, family history of HCM-related SD is accepted as a major risk marker both in AHA/ACC and European Society of Cardiology (ESC) guidelines for adults with HCM,^26^, ^27^, ^28^ albeit not considered an independent risk factor for SD in childhood by the recent Association for European Paediatric and Congenital Cardiology (AEPC) position statement.^21^ Family history of HCM-related SD occurs in about one-third of children with appropriate ICD therapy,^19^^,^^20^ and is a sole risk marker in 12% of such patients.^19^

## Risk stratification with the major marker strategy

### Prior experience

The most common approach for identifying pediatric or adult HCM patients who are candidates for ICDs to prevent SD relies on an overarching principle: ≥1 traditional risk markers judged to be major within the clinical profile of the individual HCM patient, sufficient to consider a primary prevention ICD in conjunction with shared decision making and a measure of physician judgment when necessary. This approach is endorsed by multiple expert HCM guideline panels, from the ACC and AHA since the first (2003) ACC/ESC international HCM consensus panel^29^ to the subsequent AHA/ACC guidelines in 2011^27^ and 2020,^28^ and also the PACES (Pediatric and Congenital Electrophysiology Society) 2021 Expert Consensus,^10^ endorsed by the Heart Rhythm Society, AEPC, Asia Pacific Heart Rhythm Society, the Indian Heart Rhythm Society, and the Latin American Heart Rhythm Society. The 2014 ESC HCM guidelines only recommend prophylactic ICDs for HCM children with ≥2 major markers.^26^

### Efficacy of major risk markers in children

The AHA/ACC traditional major risk marker strategy, albeit not specifically designed for children, has nevertheless proved effective and reliable for guiding informed ICD decision making in high-risk children and adults of all ages who ultimately benefit from SD prevention with prophylactic device therapy.^30^ Specifically, there are 3 studies in which> 500 children, adolescents, and young adults were prospectively assessed for increased risk based on the presence of ≥1 major risk marker,^8^^,^^19^^,^^20^ including a multicenter study with 22 institutions. In a 2-center study,^20^ sensitivity using these major risk markers was >90% for prevention of SD and specificity was about 60% for identifying patients not at risk. In addition, a single-center study^8^ demonstrated particularly high reliability of the risk markers, notably with 100% sensitivity and zero SDs (63% specificity). These data underscore certain similarities in risk stratification and ICD outcomes in children and adults with HCM. Indeed, the evolving and maturing risk stratification algorithm in HCM has contributed substantially to an overall low disease-specific mortality rate of 0.5% per year.^31^ A multicenter study from the United Kingdom confined to HCM patients ≤16 years of age reported lower sensitivity with major risk markers; however, these findings were reported in a cohort for which 70% of patients had no risk factors and the risk profile was incomplete in >25%.^32^

The current risk marker strategy endorsed by ACC/AHA also offers the flexibility to incorporate additional novel risk markers. The additive value of cardiac magnetic resonance for risk stratification was demonstrated by 2 recent studies,^33^^,^^34^ although cardiac magnetic resonance remains absent from current risk scoring algorithms.^24^^,^^35^ In addition, the individual risk marker approach creates an opportunity to apply medical reasoning and physician judgment or intuition in assigning the appropriate weight that certain risk factors deserve in individual patients, particularly important in a heterogeneous disease such as HCM. For example, evaluating syncopal episodes as a potential risk marker can be challenging in practice for determining etiology (ie, neurocardiogenic vs arrhythmic vs hemodynamic due to outflow obstruction) but is generally accepted by most observers, including the AEPC pediatric consensus panel.^21^

Of the hundreds of reported ICD implants in children and adolescents with HCM (Table 1), about 20% have been responsible for appropriate therapy terminating life-threatening ventricular tachyarrhythmias at 3% to 4% per year, a rate similar to that reported in adult HCM studies.^14^^,^^36^ Because most of these were registry studies reporting outcomes in young high-risk HCM patients implanted with ICDs, the number of SD events that occurred in patients without ICDs is unknown, prohibiting a valid calculation of true sensitivity.

**Table 1:** Children and adolescents with HCM and reported primary and secondary prevention of sudden death with ICDs

Reference citation	HCM implantations	ICD appropriate therapy	Appropriate therapy (%)	Age at implantation (mean/median) (y)	Average years to first therapy	Implantation indication
Silka et al, 1993^50^	44	NA	NA	14.5	1	NA
Kaski et al, 2007^51^	22	4	18	14	3.3	Primary: 17 Secondary: 5
Decker et al, 2009^52^	17	1∗	6	NA	NA	Primary: 13 Secondary: 4
Maron et al, 2013^19^	224	43	19	14	3	Primary: 188 Secondary: 36
Kamp et al, 2013^53^	73	11	15	14.8	1.7	Primary: 61 Secondary: 12
Ziółkowska et al, 2015^54^	21	5	24	NA	NA	Primary: 15 Secondary: 6
Bharucha et al, 2015^55^	13	4	31	NA	NA	Primary: 13 Secondary: 0
Maron et al, 2016^20^	231†	42∗	18	21	2	Primary: 212 Secondary: 19
Dechert et al, 2016^56^	24	3	13	12.3	2	Primary: 23 Secondary: 1
Östman-Smith et al, 2017^57^	NA	3	NA	NA	NA	NA
Ho et al, 2018^58^	59	5	8	NA	NA	Primary: 51 Secondary: 8
Maurizi et al, 2018^5^	17	5	29	16	NA	Primary: 7 (sic) Secondary: 12 (sic)
Winkler et al, 2018^9^	14	3	21	NA	NA	NA
Norrish et al, 2019^24^	267	24	9	NA	NA	Primary: 244 Secondary: 23
Norrish et al, 2019^42^	135	19	14	NA	NA	Primary: 108 Secondary: 27
Norrish et al, 2019^32^	110	12	11	NA	NA	NA
Balaji et al, 2019^6^	446	55	12	Primary: 14.6 Secondary: 14.7	Primary: 3.0 Secondary: 1.1	Primary: 349 Secondary: 97
Maron et al, 2019^30^	NA	10	NA	NA	6.7	Primary: 10 Secondary: 0
Miron et al, 2020^35^	125	14	11	NA	2.2	Primary: 102 Secondary: 23
Rowin et al, 2020^8^	60†	9∗	15	15	4	Primary: 60 Secondary: 0
Rowin et al, 2020^14^	NA	12	NA	15.3	8.2	NA
Edelson et al, 2020^59^	214	NA	NA	NA	NA	NA
Norrish et al, 2021^60^	10‡	2∗	20	12 and 2.5	NA	Primary: 6 Secondary: 3
Norrish et al, 2021^17^	90†^,^‡	25∗	28	13	NA	Primary: 67 Secondary: 23
Tunca Sahin et al, 2021^61^	38	9	24	NA	NA	Primary: 35 Secondary: 3
Alashi et al, 2021^62^	23†	3	13	NA	NA	Primary: 23 Secondary: 0
Norrish et al, 2022^25^	NA	16	NA	NA	NA	NA
Norrish et al, 2022^63^	332†^,^‡	38	11	Primary: 7 Secondary: 14	Primary: 4.8 Secondary: 3	Primary: 291 Secondary: 31
Chan et al, 2022^64^	9†	3	33	12	NA	Primary: 8 Secondary: 1
Silvetti et al, 2022^65^	17	7	41	12	NA	Primary: 14 Secondary: 3
Ali et al, 2022^33^	11	NA	NA	NA	NA	Primary: 11 Secondary: 0

HCM = hypertrophic cardiomyopathy; ICD = implantable cardioverter-defibrillator; NA = not available.

∗ Includes ≥2 appropriate therapies.

† Includes implants <13 years.

‡ Includes some ICDs from other studies from the same authors.

On the other hand, device failures in HCM leading to SD have been exceedingly rare. Perhaps the most visible such event involved a young high-risk nonobstructive HCM patient implanted with an ICD that proved unreliable for terminating VF due to a short-circuiting defect known only to the manufacturer.^37^ This situation, known as the Guidant Affair, not only led to the recall of >200,000 implantable devices, but also triggered greater transparency between the device industry, patients, and managing physicians.

## Emergence of predictive risk scoring models designed for children with HCM

Alternate risk prediction strategies have emerged with a novel statistical model for identifying high-risk HCM children that generates 5-year SCD estimates after imputation of disease-related features into a multivariate logistic regression formula.^24^, ^25^, ^26^^,^^38^ Essentially, this method collects and enters data into a complex equation that weighs each component.

The first of these prediction models with the largest experience in children is HCM Risk-Kids,^24^ which is similar to the adult-based ESC-SD risk score,^26^^,^^38^ but with some differences in incorporated variables (ie, age and family history of SD are excluded, while left atrial diameter and maximal LV wall thickness are expressed as *Z* scores) (Table 2). HCM Risk-Kids classified all patients into 1 of 3 risk categories based on the estimated 5-year SCD rate—low (<4%), intermediate (4%–6%), or high risk (≥6%)—but unlike the ESC risk score version did not attach specific recommendations for ICD implants to these categories.^24^^,^^25^ The HCM Risk-Kids score was endorsed by 2022 ESC guidelines for ventricular arrhythmias and the prevention of SD.^39^

**Table 2:** Comparison of 3 strategies for sudden death prevention in children with HCM

Variable	ACC/AHA risk markers^27^	HCM Risk-Kids^24^	PRIMaCY^35^
ICD decisions	≥1 risk marker	Quantitative score	Quantitative score
Tested prospectively	+	0	0
Annual event rate	Not used	5 y	5 y
Age of eligible patients	<21 y	<16 y	<18 y
Flexible algorithm	+	0	0
Risk markers used			
Syncope	+	+	+
VS thickness	0	+ (*Z* score)	+ (*Z* score)
Max LV wall thickness	+	0	0
PW thickness	0	0	+ (*Z* score)
LA diameter	0	+ (*Z* score)	+ (*Z* score)
LVOT gradient	0	+	+
NSVT	+	+	+
FH of HCM-SD	+	0	0
LGE on MRI	+	0	0
LV apical aneurysms	+	0	0
End stage (LVEF <50%)	+	0	0
Age	+	+	+
Genotype	0	0	+

A plus sign (+) indicates present; a zero indicates absent.

ACC = American College of Cardiology; AHA = American Heart Association; FH = family history; HCM = hypertrophic cardiomyopathy; ICD = implantable cardioverter-defibrillator; LA = left atrial; LGE = late gadolinium enhancement; LV = left ventricular; LVEF = left ventricular ejection fraction; LVH = left ventricular hypertrophy; LVOT = left ventricular outflow tract; MRI = magnetic resonance imaging; NSVT = nonsustained ventricular tachycardia; PRIMaCY = PRecIsion Medicine for CardiomyopathY; PW = posterior wall; SD = sudden death; VS = ventricular septum.

PRIMaCY (PRecIsion Medicine for CardiomyopathY) is a separate pediatric-specific North American retrospective multicenter model published after the larger HCM Risk-Kids. It is composed of 572 patients <18 years of age and validated using data from the SHaRe (Sarcomeric Human Cardiomyopathy Registry) registry.^35^ The C-statistic for risk prediction is 0.71 to 0.75 for the developmental and external validation cohort and was improved by incorporating the LV posterior wall thickness *Z* score and genetic status into the risk assessment algorithm. Such segmental hypertrophy is a particularly unusual variable not previously incriminated in SD risk in HCM at any age. Also, reporting the absence of LV outflow obstruction as an independent risk marker in PRIMaCY is not consistent with any prior risk stratification data reported in HCM populations of any age. The consensus AEPC panel noted weaknesses in PRIMaCY: confidence intervals for performance estimates were not reported and the final equation was not available; it was recommended that comparison of PRIMaCY with HCM Risk-Kids was required to assess its performance in clinical practice.^21^

### Assessment of quantitative risk scoring

The primary statistical metric employed to judge performance of any risk scores, eg, ESC,^38^ Risk-Kids,^24^ or PRIMaCY,^35^ begins with concordance (ie, the C-statistic), measuring the area under the curve. Pediatric HCM risk scores demonstrate moderate discriminatory power (0.69–0.76) in differentiating patients who experienced a SD event vs patients who did not. However, the C-statistic is a group model that does not provide cutpoints for clinical decision making that would be applicable to individual patients.

**Figure 1:**
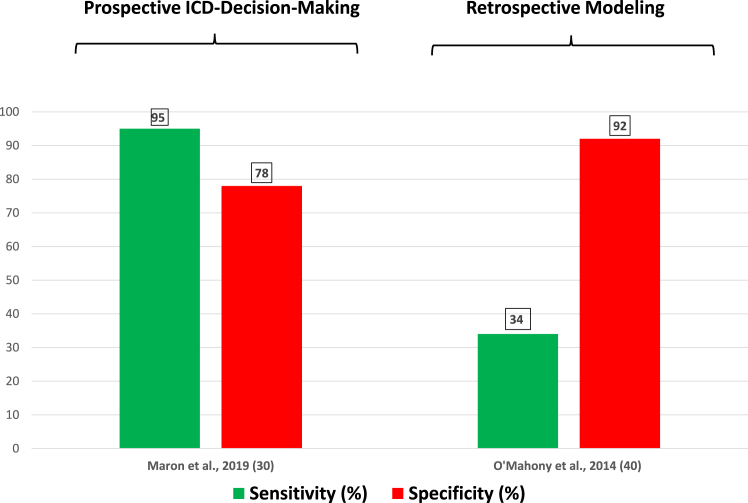
Comparison of sensitivity and specificity obtained with prospective American Heart Association/American College of Cardiology traditional risk markers and retrospective risk stratification strategies with risk scoring proposed for prevention of sudden death in *adults* with hypertrophic cardiomyopathy. For risk scoring, sensitivity and specificity are based on retrospective theoretical application of the risk score (≥6% per 5 years) to an established cohort with implantable cardioverter-defibrillators (ICDs) that has already been risk stratified by traditional risk markers.

A retrospective analysis of the patient cohort from the HCM Risk-Kids study (n = 527)^24^ and an independent validation study (n = 421) reported^25^ a calculated sensitivity for young patients at risk for subsequent SD events of 74% to 77%, at an average age of 11 to 12 years (albeit with the major limitation of 30%–50% missing data). This sensitivity is substantially lower than that achieved with the prospective risk marker approach in children (>90%)^8^^,^^20^ or in adult high-risk HCM patients (95%) (Figure 1).^30^ Such differences in reported sensitivity are particularly relevant in pediatrics, and potentially extrapolate to greater numbers of missed opportunities.

**Figure 2:**
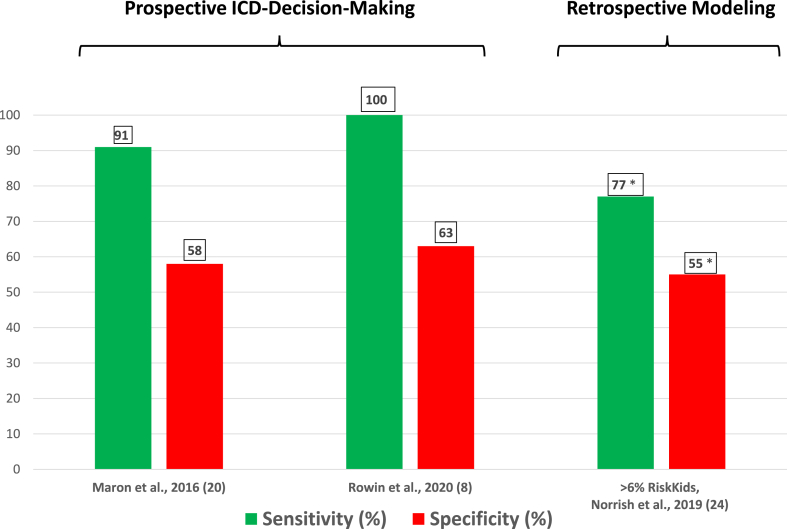
Comparison of sensitivity and specificity obtained with prospective American Heart Association/American College of Cardiology traditional risk markers and retrospective risk stratification strategies with risk scoring proposed for prevention of sudden death in *children and adolescents* with hypertrophic cardiomyopathy. ∗For risk scoring, sensitivity and specificity are based on retrospective theoretical application of the risk score (≥6% per 5 years) to an established cohort with implantable cardioverter-defibrillators (ICDs) that has already been risk stratified by traditional risk markers.

The hypothetical sensitivity for preventing SD in the retrospective HCM Risk-Kids study was calculated by first defining a cohort of 527 children with HCM already-stratified by risk markers.^25^ The HCM Risk-Kids score (cutoff ≥6% per 5 years to define high-risk) was then applied to outcomes with or without ICDs to test efficacy of the model (Figure 2). A wider hypothetical cutoff ≥4% for 5 years was associated with a higher calculated sensitivity of 91%. Notably, the annual mortality rate predicted between the lowest and highest risk score is only 0.4%, a limitation raising a potential interpretation dilemma for parents attempting to make decisions regarding ICDs, an issue similar to that previously identified in using the ESC score for adult HCM patients.^40^

The relatively high specificity for identifying patients not at risk of SD attributed to the ESC scoring systems previously in adults^26^^,^^38^^,^^40^ suggested that this strategy could modestly reduce the numbers of implantations in lower-risk patients, thereby potentially mitigating excessive ICD use. However, specificity of the HCM Risk-Kids model is in the range of 35% to 55%,^25^ which, if implemented in real-world clinical practice, would likely result in many devices that do not deliver appropriate therapy over the follow-up (Figure 2). Indeed, the specificity of HCM Risk-Kids is lower than that encountered in adults with HCM risk stratified by the ESC risk score.^25^^,^^38^ In comparison, the specificity of the traditional risk marker strategy is 58% to 63%,^8^^,^^20^ representing a somewhat lesser degree of ICD overtreatment (Figure 2).

**Figure 3:**
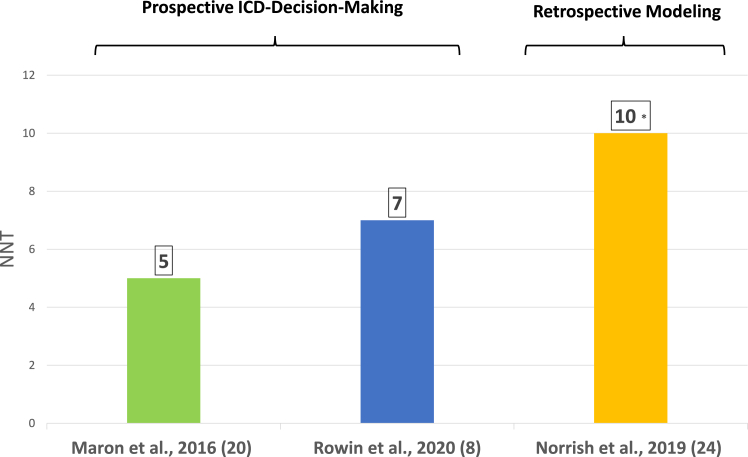
Number of patients needed to treat (NNT) with ICDs to prevent 1 sudden death event shown for prospective and retrospective modeling studies. ∗As in Figure 2, the NNT is based on retrospective theoretical application of the risk score to an established cohort with implantable cardioverter-defibrillators (ICDs) that has already been risk stratified by traditional risk markers.

Notably, when specificity is judged as the number of ICDs necessary to implant for each potential lifesaving therapy, the traditional risk marker strategy in children is associated with a lower number needed to treat of 5 to 7 compared with 10 for the HCM Risk-Kids scoring tool (Figure 3). It should also be underscored that in HCM ICD excess implied by the relatively low specificity reported for both these strategies does not necessarily represent unnecessary implantations, as first ICD therapy is often significantly delayed (ie, ≥10 years after implantation in one-third of HCM patients).^14^

## Potential strengths and limitations of quantitative risk prediction scores

**Figure 4:**
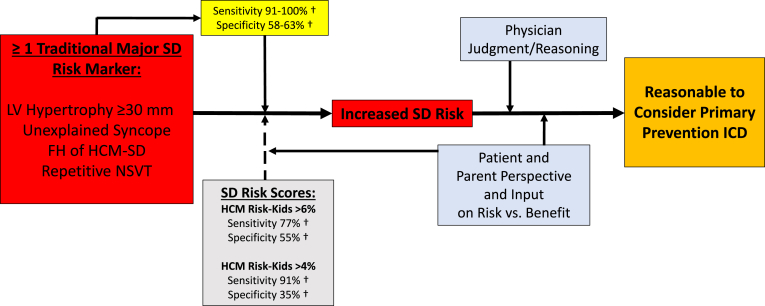
Risk stratification decision-making algorithm for primary prevention implantable cardioverter-defibrillator (ICD) implants in high-risk children and adolescents with hypertrophic cardiomyopathy (HCM). Family history (FH) of HCM-related sudden death (HCM-SD) indicates ≥1 HCM-related sudden death. LV = left ventricular; NSVT = nonsustained ventricular tachycardia. †From Maron et al^20^, Rowin et al^8^ and Norrish et al^24^.

The strength of risk scoring literature has been the large pediatric populations studied.^24^^,^^35^ The application of such scoring models to clinical practice, for estimating the level of SD risk over 5 years as low, intermediate, or high, could potentially clarify ICD decision making for young patients and their parents (Figure 4). This strategy of using online calculators could mitigate uncertainty surrounding risk stratification in pediatrics, a potential advantage given the complexities associated with ICDs in this unique patient group.

However, the 5-year estimates can also be challenging for parents in interpreting long-range aspirations for their young children (ie, it may be difficult to integrate risk scores into ICD decisions by translating short-term calculations to extended periods of life, particularly since HCM itself is compatible with normal longevity).^41^

Finally, in these scoring studies, selection of patients for ICDs was not made prospectively in cohorts for which outcome and ICD efficacy was determined. Without such outcome data, the reliability of risk scoring systems to predict future events and prevent SD remains incompletely resolved at this time. Without comparative study evidence, the AEPC consensus panel nevertheless concluded that HCM Risk-Kids outperformed the AHA/ACC major risk marker strategy.^21^

## Worldwide experience with SD prevention in children

The global use of ICDs for high-risk pediatric HCM patients is summarized in Table 1. The published literature reports >2000 children, adolescents, and young adults implanted with ICDs over >20 years for primary (∼75%) or secondary (∼25%) prevention. Notably, virtually all these prior risk stratification decisions selecting patients for ICD implantations were based on the traditional major risk factors (Table 3) that were evident in the available guidelines.^29^ Of note, there is a paucity of data concerning the efficacy of ICD implants in children with HCM under the age of 10 years, who are rarely candidates for primary prevention in clinical practice.^24^^,^^42^

**Table 3:** Prospective studies of sudden death prevention in children and adolescents with hypertrophic cardiomyopathy using American College of Cardiology/American Heart Association major risk marker strategy

Study	Mean age at implantation (y)	No. implants	No. ICD appropriate therapy	No. primary prevention implants	No. secondary prevention implants	Strengths	Countries	Sensitivity (%)∗
Maron et al, 2013^19^	14	224	43	188	36	Prospective; multicenter; 22 institutions†	United States, Canada, Europe, and Australia	NA
Maron et al, 2016^20^	21	231	42	212	19	Prospective	United States	91
Rowin et al, 2020^8^	15	60	9	60	0	Prospective	United States	100

ICD = implantable cardioverter-defibrillator; NA = not available.

∗ For the purpose of sensitivity and specificity analyses, true positives are defined as individuals who received recommendations for ICDs and subsequently experienced sudden death events; false positives as patients who received recommendations for ICDs but did not subsequently experience a sudden death event, false negatives as patients who did not receive recommendations for ICDs but subsequently experienced sudden death events; and true negatives as patients who did not receive recommendations for ICDs nor experience a sudden death event. Sensitivity was calculated using the following formula true positives / (true positives – false negatives).

† Italy, Greece, Spain, Germany, France, Czech Republic.

## Future needs and perspectives

Although selection of high-risk patients of all ages for ICD implantations has evolved, nevertheless excess implants in young patients who do not experience appropriate therapy represent a concern for the pediatric population, given the risks for device-related complications over decades. Therefore, advancing the precision of risk stratification by increasing the specificity of implants for patients (but not at the expense of sensitivity) is a priority for both children and adults with HCM. This could be addressed by assembly of prospective data in large pediatric HCM populations using AHA/ACC traditional risk markers or risk scoring strategies. It is possible that innovative initiatives such as network medicine^43^^,^^44^ or artificial intelligence^45^ will provide clarity to this area. In addition, novel risk markers may emerge from properly designed prospective studies in large populations with and without ICDs.

Finally, the unique circumstances surrounding chronic device implantations in young patients underscores the need for technical ICD advances specifically tailored toward the pediatric population: smaller generator size appropriate for children, improved defibrillator lead longevity and integrity, and pediatric algorithms to reduce inappropriate shocks. By virtue of addressing long-term complications inherent in transvenous devices, subcutaneous lead systems have undergone several modifications and improvements over the last decade directed toward efficacy and safety.^46^^,^^47^ Improvements in the implantation procedure (including default adoption of intermuscular device placement and 2-incision technique) are mitigating perioperative complications^46^^,^^47^ increasingly making the subcutaneous ICD an option to avert long-term lead complications in pediatric patients. Future ICD systems are likely to be minimally invasive, nontransvenous, and leadless, avoiding the attendant risks of lead failure and need for risky extraction procedures.

## Conclusion

The ICD is a major advance in the management of HCM shown to reliably abort lethal ventricular tachyarrhythmias by defibrillation shocks and antitachycardia overdrive pacing and thereby prevent SDs in HCM patients of all ages, including children and adolescents. Nevertheless, recommending potentially lifelong ICD therapy to very young patients represents a challenging clinical situation within cardiology (ie, differing in many respects from that encountered in the high-risk adult HCM population), particularly given the asymptomatic and healthy-appearing patients and the unavoidable chronic and probably lifelong commitment for implants with the potential for device-related complications.

However, aspirations for a perfect risk stratification strategy for patients with a heterogeneous disease such as HCM associated with a low SD event rate is unrealistic. It is most productive to consider risk stratification for pediatric HCM patients in terms of a weighted balance, with greater emphasis on identifying at-risk patients (sensitivity) at the expense of potentially incurring device-related complications and inconvenience in patients who ultimately may not experience appropriate therapy (specificity).^48^ Although risk stratification approaches can differ based on commitment to a particular societal guideline, cost-benefit analyses, or country of residence, the core principle that is fundamental to all strategies remains the recognized risk markers (Figure 4).

The AHA/ACC traditional major risk marker approach supported by guidelines and based on prospective real-world ICD data is associated with high sensitivity for detection of at-risk patients. Individualized SD risk scores designed specifically for pediatric patients to estimate risk for SD are potentially useful in facilitating physician-patient (family) interactions surrounding risk vs benefit for long-term device therapy. In this regard, it is reasonable to consider pediatric risk scores in a similar fashion as the approach taken with adult risk scores in the recent 2020 AHA/ACC guidelines for HCM.^28^ In that panel, scoring initiatives were recommended as an adjunctive tool to complex ICD decisions by providing additional information related to the magnitude of risk.

Finally, it is important to recognize an understandable reticence on the part of pediatricians and other clinicians to obligate healthy appearing young patients to lifelong device implants. However, to underutilize the ICD in high-risk children and adolescents could represent a lost opportunity to elicit the power of this transformative and lifesaving technology now available for over 30 years,^49^ capable of delivering the long-standing aspiration of reducing SD events in HCM once considered the most common cause of SD in the young.
